# Correction: Promoter choice influences alternative splicing and determines the balance of isoforms expressed from the Mouse *bcl-X* gene

**DOI:** 10.1016/j.jbc.2021.100688

**Published:** 2021-04-28

**Authors:** Adali Pecci, Luciana Rocha-Viegas, José Lino Barañao, Miguel Beato

VOLUME 276 (2001) PAGES 21062–21069

The second author of the above article is incorrectly identified as “Luciana Rocha Viegas.” The correct name is “Luciana Rocha-Viegas.”

